# End-to-End, Pixel-Wise Vessel-Specific Coronary and Aortic Calcium Detection and Scoring Using Deep Learning

**DOI:** 10.3390/diagnostics11020215

**Published:** 2021-02-02

**Authors:** Gurpreet Singh, Subhi J. Al’Aref, Benjamin C. Lee, Jing Kai Lee, Swee Yaw Tan, Fay Y. Lin, Hyuk-Jae Chang, Leslee J. Shaw, Lohendran Baskaran, on behalf of the CREDENCE and ICONIC Investigators

**Affiliations:** 1 Dalio Institute of Cardiovascular Imaging, Weill Cornell Medicine, New York, NY 10021, USA; gurpreets.nanda@gmail.com (G.S.); sjaref@gmail.com (S.J.A.); bcl2004@med.cornell.edu (B.C.L.); fal9003@med.cornell.edu (F.Y.L.); les2035@med.cornell.edu (L.J.S.); 2 Department of Radiology, New York-Presbyterian Hospital, New York, NY 10021, USA; 3 Department of Radiology, Weill Cornell Medicine, New York, NY10021, USA; 4 Data Science Capabilities, Global Vx Tech, GlaxoSmithKline, Collegeville, PA 19426, USA; 5 Division of Cardiology, Department of Medicine, University of Arkansas for Medical Sciences, Little Rock, AR 72205, USA; 6 Department of Cardiovascular Medicine, National Heart Centre, Singapore 169609, Singapore; jklee9993@gmail.com (J.K.L.); tan.swee.yaw@singhealth.com.sg (S.Y.T.); 7 Division of Cardiology, Severance Cardiovascular Hospital, Integrative Cardiovascular Imaging Center, Yonsei University College of Medicine, Seoul 03722, Korea; hjchang@yuhs.kr

**Keywords:** coronary artery calcium, deep learning, machine learning

## Abstract

Conventional scoring and identification methods for coronary artery calcium (CAC) and aortic calcium (AC) result in information loss from the original image and can be time-consuming. In this study, we sought to demonstrate an end-to-end deep learning model as an alternative to the conventional methods. Scans of 377 patients with no history of coronary artery disease (CAD) were obtained and annotated. A deep learning model was trained, tested and validated in a 60:20:20 split. Within the cohort, mean age was 64.2 ± 9.8 years, and 33% were female. Left anterior descending, right coronary artery, left circumflex, triple vessel, and aortic calcifications were present in 74.87%, 55.82%, 57.41%, 46.03%, and 85.41% of patients respectively. An overall Dice score of 0.952 (interquartile range 0.921, 0.981) was achieved. Stratified by subgroups, there was no difference between male (0.948, interquartile range 0.920, 0.981) and female (0.965, interquartile range 0.933, 0.980) patients (*p* = 0.350), or, between age <65 (0.950, interquartile range 0.913, 0.981) and age ≥65 (0.957, interquartile range 0.930, 0.9778) (*p* = 0.742). There was good correlation and agreement for CAC prediction (rho = 0.876, *p* < 0.001), with a mean difference of 11.2% (*p* = 0.100). AC correlated well (rho = 0.947, *p* < 0.001), with a mean difference of 9% (*p* = 0.070). Automated segmentation took approximately 4 s per patient. Taken together, the deep-end learning model was able to robustly identify vessel-specific CAC and AC with high accuracy, and predict Agatston scores that correlated well with manual annotation, facilitating application into areas of research and clinical importance.

## 1. Introduction

Vascular calcification is strongly predictive of cardiovascular morbidity and mortality [1]. Gated non-contrast cardiac computed tomography (CT) is the most established tool in the assessment of coronary artery calcium (CAC), and its quantification uses the well-established Agatston method to derive a CAC score (CACS) [2].

However, this method has inherent shortcomings. It is unable to distinguish between the regional features of multivessel disease, such as density, diffusivity (the degree of dispersion of CAC within the coronary tree), and distribution (location and number of coronary arteries involved) that may, as well as the presence of aortic calcium (AC), carry independent prognostic value and improve risk prediction by an additional 5–20% [1]. Furthermore, evaluation of these features requires laborious manual annotation, limiting the utility of these features when used on a large number of scans.

Because it is derived as a summation, CACS is unable to accurately convey more nuanced, yet significant findings, such as differentiating between higher and lower density CAC, or lesion location, distribution, shape, and size [3]. This aggregation on lesion, vessel, and patient levels results in information resolution loss when transiting from the acquired image to the final score, leading to seemingly divergent prognostic implications of a high or low CACS [4]. As such, the final information yield is lower than is possible.

Machine learning (ML) is a field that proposes novel algorithmic strategies for the construction of predictive data-driven models from large datasets [5]. Deep learning is a subdomain that utilizes more sophisticated frameworks with the ability to perform automated feature extraction and excels at modeling extremely complicated non-linear relationships between inputs and outputs. It can substantially outperform systems that rely on features supplied by domain experts [6]. In this study, we develop, demonstrate, and evaluate an end-to-end, rapid deep learning model for the rapid identification of vessel-specific CAC and AC on a pixel-wise level from gated noncontrast cardiac CTs.

## 2. Materials and Methods

### 2.1. Study Population

The study population comprised a convenience aggregation of two international, multicenter, prospective, observational registries, that have been described in detail elsewhere [7,8]. In brief, patients that underwent clinically indicated coronary computed tomography angiography (CCTA) were included, with prospectively collected history, risk factors, and symptoms at baseline. Inclusion criteria were patients undergoing clinically indicated CCTA and aged ≥ 18 years of age. Exclusion criteria were known coronary artery disease (CAD), hemodynamic instability, inability to provide consent, pregnancy, known adult congenital heart disease, baseline irregular heart rhythm, heart rate ≥ 100 beats per minute, systolic blood pressure ≤ 90 mmhg, contraindications to beta-blockers or nitroglycerin or adenosine, and uninterpretable CCTA. Each site obtained local institutional review board or ethics board approval. This resulted in a total of 846 patients. Those with missing or uninterpretable CT CAC images were excluded, resulting in a total of 377 patients for this study.

### 2.2. Image Acquisition and Identification of CAC

**Figure 1:**
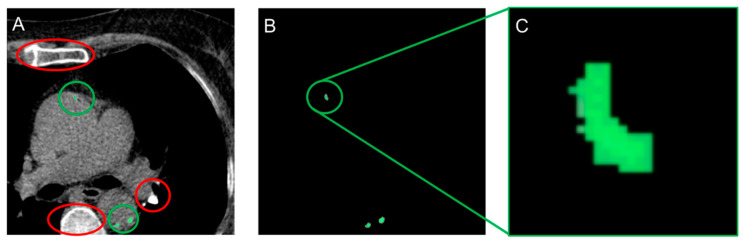
(**A**) Annotation in DICOM format requires differentiation between several non-cardiac structures with HU > 130 such as the vertebrae and sternum (red), and cardiac calcium (green). (**B**) and ×15 magnification, (**C**) The colored annotation in DICOM format is extracted using color-based K-means clustering. Abbreviations: DICOM = Digital Imaging and Communications in Medicine, HU = Hounsfield units.

Imaging data were acquired using multi-detector row CT scanners consisting of 64-rows or greater. Image acquisition, image post-processing, and data interpretation were performed according to guidelines [9]. Gated non-contrast cardiac scans were performed with triggering corresponding to 60–80% of the RR interval. Images were obtained and reconstructed at 2.5- or 3.0-mm intervals beginning one centimeter below the carina and progressing caudally to include all coronary arteries. Both CAC and AC within the field of view were separately identified and annotated (Figure 1). Images were in Digital Imaging and Communications in Medicine (DICOM) format. AC was a combination of aortic valve calcification (AVC) and thoracic aortic calcification (TAC). The coronaries were divided into left anterior descending (LAD), right coronary artery (RCA) and left circumflex (LCx). Annotation was done at a core laboratory blinded to all other data. These annotations established and verified by board-certified cardiologists (L.B., 5 years of training; S.A., 3 years of training), were used as the ‘ground truth’ for the deep learning model. CACS and AC were scored using the Agatston method [2].

### 2.3. Splitting of Dataset and Preprocessing

The entire dataset containing 377 patients was split into three parts; training (60%, 226 patients, 19, 543 images), testing (19%, 72 patients, 4443 images), and validation (21%, 79 patients, 5111 images). The splitting of the dataset aimed at maintaining a ratio of 60:20:20 (Train: Test: Validation). The process of extraction of the ground truth from the output was accomplished using K-means clustering (Figure 1). All scans were converted to Hounsfield Units (HU) and resampled to a voxel spacing of 1 × 1 × 3 mm.

### 2.4. Deep Learning Model

**Figure 2:**
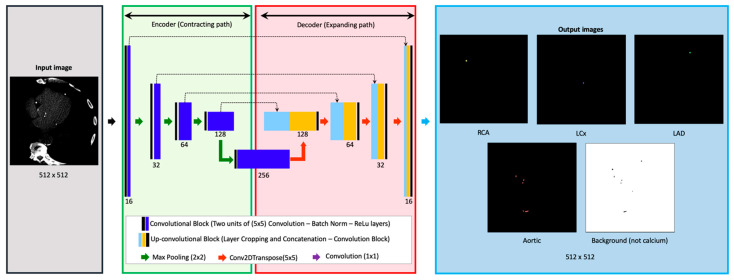
The deep learning model architecture. An input image of 512 × 512 is fed as input to the model (gray). The model used in this study was inspired by U-Net and has two main parts; the encoder (green) and the decoder (red). In the encoder, starting from the initial number of filters as 16, the number of filters was doubled after each subsequent convolutional block followed by a max pooling operation. The decoder part restores the dimensionality of the segmentation mask by concatenating the features from the encoder by application of transposed convolutional operations followed by convolutions, batch normalization and ReLu operations. The segmentation mask of the same resolution is obtained as the model output (blue). Abbreviations: LAD = left anterior descending, LCx = left circumflex, RCA = right coronary artery.

The CNN architecture used in this study was inspired by the U-Net framework for semantic segmentation (Figure 2) [10]. The architecture comprised two main subparts: (i) a contracting path (Encoder) and (ii) an expansive path (Decoder). After each convolutional block, the number of filters was doubled starting with 16 filters in the first layer. The expansive path is composed of four upsampling blocks. In the final layer, a (1 × 1) convolutional layer with softmax activation was applied. All convolutional layers were initialized with He-uniform initializations unless mentioned otherwise [11]. The architecture was retrofitted to incorporate the nuances of accomplishing segmentation of vascular calcification. First, the problem was posed as an n+1 class problem to the architecture where n was the number of classes trained for performing segmentation. An additional class was added to account for the background. Secondly, to account for imbalance between the higher number of images with AC than CAC, undersampling was performed, keeping in mind that images from the same patients always belonged to only one of the sets—i.e., training, testing, or validation. Thirdly, since there existed class imbalance, the loss function was also weighted to account for the importance of CAC to be suited for clinical application. Fourthly, the location and the size of the CAC was highly correlated in the 3D volume, owing to the fact that the CAC tends to be much smaller in volume compared to AC. To adapt to this, exhaustive data augmentation was performed. Lastly, unlike many datasets with natural image segmentation, CT images posed unique challenges such as the need to convert these images into HU, the inability to apply z-score-like normalization by directly taking maximum and minimum for an image, and the need for the data augmentation to replicate the naturally occurring defects. The proposed approach was conceived by iteratively eliminating the abovementioned unique challenges.

### 2.5. Model Evaluation

**Figure 3:**
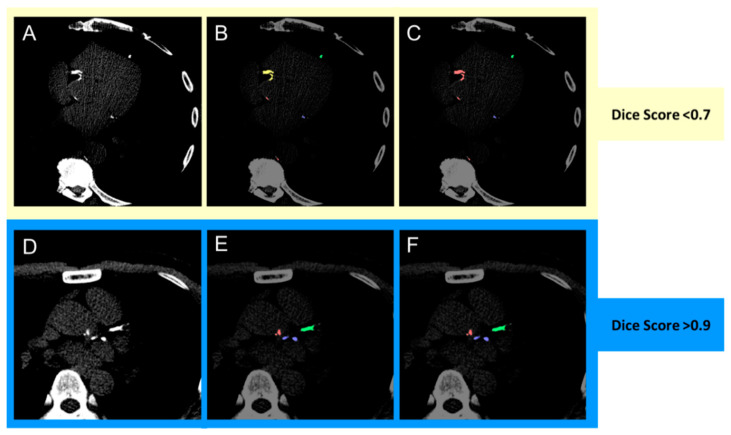
Dice score visualization. (**A**,**D**) Original image; (**B**,**E**) ground truth annotation; (**C**,**F**) deep learning model output. The Dice score is commonly used to gauge the model performance and ranges from 0 to 1, with 1 corresponding to a pixel perfect pixel match between the ground truth annotation. In the images above, the prediction with the lower Dice score (**C**) is due to the model misclassifying right coronary artery calcification (yellow) as aortic calcium (red). The prediction with the higher Dice score (**F**) correctly classifies left anterior descending (green), left circumflex (blue) and aortic calcification (red) correctly.

The image-based performance metric used Dice loss as the performance metric [12]. The Dice similarity score quantifies the pixel-wise degree of similarity between the model predicted segmentation mask and the ground truth (Figure 3). It ranges from 0–1, with 1 denoting that the predicted mask is identical to the ground truth, expressed as

(1) $$\mathrm{Dice}\;\mathrm{similarity}\;\mathrm{coefficient}=\;\frac{\left(2\times \mathrm{True}\;\mathrm{Positive}\right)}{\left[2\times \left(\mathrm{True}\;\mathrm{Positive}+\mathrm{False}\;\mathrm{Positive}+\mathrm{False}\;\mathrm{Negative}\right)\right]}$$

### 2.6. Statistical Analysis

Continuous and normally distributed variables were expressed by their mean ± standard deviation or median with the interquartile range as appropriate and categorical data by their number and percentage. Wilcoxon rank-sum test was conducted to examine the difference in Dice scores by sex and age subgroups. Levels of agreement between the model prediction and ground truth (manual annotation) were assessed on the test set. Correlations between model prediction and ground truth were assessed with the nonparametric Spearman’s rank test of correlation, without the normality assumption.

## 3. Results

The dataset comprised 377 patients, containing 24,507 images. The cohort mean age was 64.2 ± 9.8 years, and 33.16% were female. Prevalence of diabetes, dyslipidemia, hypertension, and smoking was 29.71%, 56.50%, 64.19%, and 32.36% respectively. LAD, RCA, LCx, triple vessel, and aortic calcification were present in 74.87%, 55.82%, 57.41%, 46.03% and 85.41% of patients respectively. Prevalence of CACS of 0, 1–99, 100–399 and ≥400 were 11.39%, 11.39%, 24.05 % and 53.16% respectively. (Table 1). Overall median CACS was 560 ± 1063. There was no difference between male (603 ± 1200) and female (473 ± 707) (*p* = 0.187), but CACS for age <65 (450 ± 909) trended towards being lower than for age ≥65 (658 ± 1176) (*p* = 0.054). The prevalence of AC > 0 was 33.42%. The overall median AC score was 2000 ± 4368.

**Figure 4:**
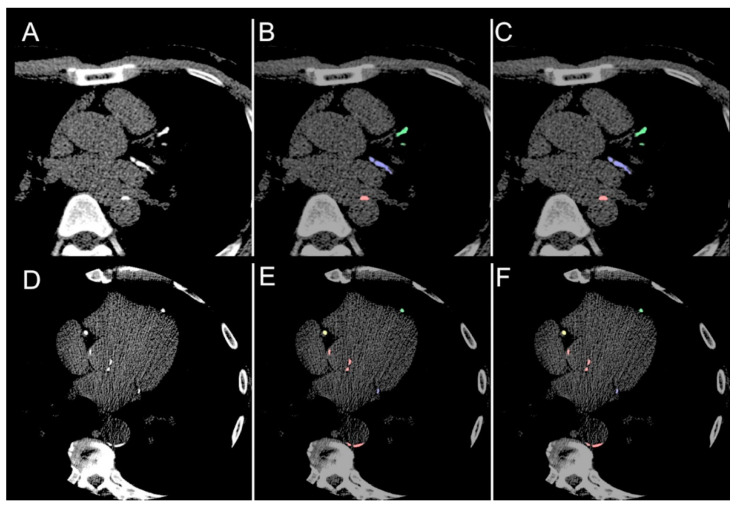
Comparison. (**A**,**D**) Illustrative comparisons between original image; (**B**,**E**) manual annotation ground truth; (**C**,**F**) the model predicted segmentation mask. The model is able to identify both calcifications of the left anterior descending (green), left circumflex (blue) and right coronary arteries (yellow) as well as aortic calcium (red).

For CAC (LAD, RCA, and LCx), the overall Dice score was 0.952 (interquartile range 0.921, 0.981), with no significant difference between the scores for male (0.948, interquartile range 0.920, 0.981) and female (0.965, interquartile range 0.933, 0.980) patients (*p* = 0.350) or between age <65 (0.950, interquartile range 0.913, 0.981) and age ≥65 (0.957, interquartile range 0.930, 0.9778) (*p* = 0.742). Overall Dice scores for the LAD, RCA. LCx and AC were 0.971 (interquartile range 0.930, 1.000), 0.963 (interquartile range 0.889, 0.991), 0.955 (interquartile range 0.894, 1.000) and 0.832 (interquartile range 0.759, 0.897) respectively, with no significant difference between sex- or age-stratified subgroups (Table 2). Figure 4 demonstrates comparisons between the ground truth and model predictions. Automated segmentation took approximately 4 ± 0.93 s per patient, at 0.06 s per slice, whereas manual segmentation took approximately 2 min per patient.

**Figure 5:**
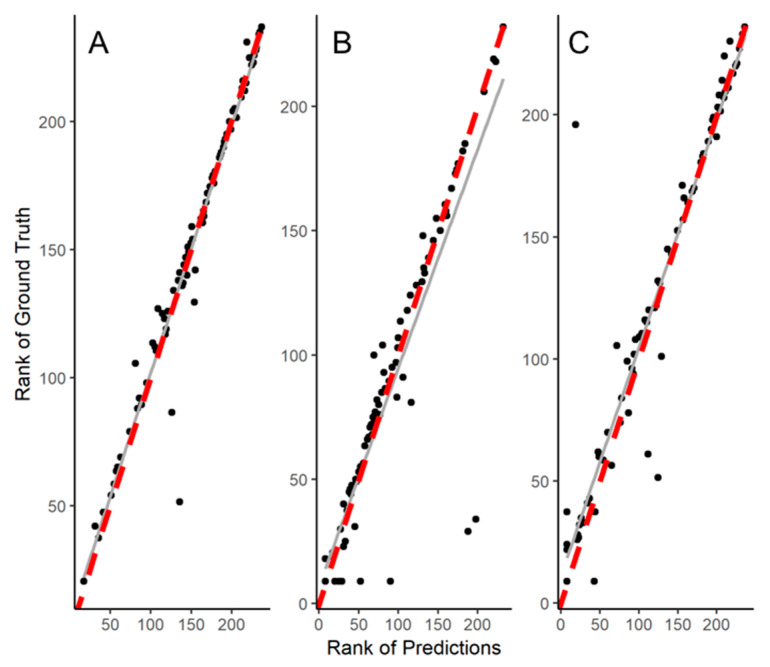
Correlation. Linear regression plots for the differences and agreement between deep learning predicted Agatston scores and the ground truth manually annotated scores for (**A**) total cardiac calcium, (**B**) coronary artery calcium, and (**C**) aortic calcium.

**Figure 6:**
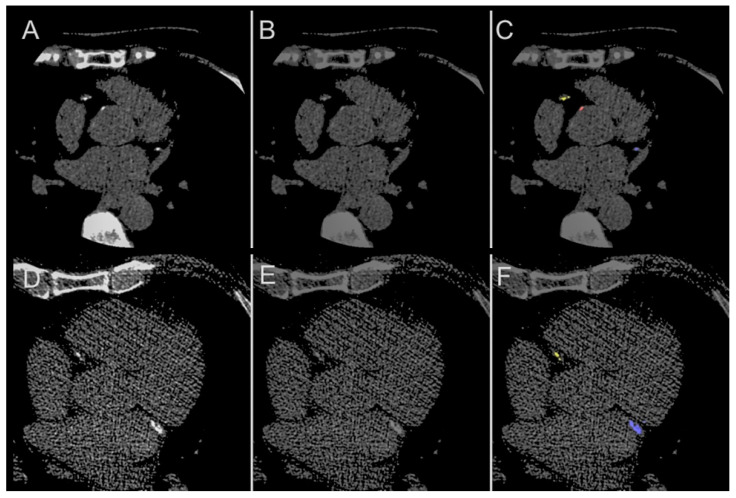
Potential for Error Detection. Illustrative examples of the model as a ‘second reader’, correcting the human error. (**A**,**D**) The original images showing calcification of the left circumflex (blue) and right coronary arteries (yellow), as well as aorta (red); (**B**,**E**) manual annotation, showing missed calling of calcification; (**C**,**F**) correct identification of calcifications that were missed by manual annotation.

Linear regression plots for the ranking of predictions against ranking of ground truth are shown (Figure 5). There was good correlation and agreement for CAC prediction (rho = 0.876, *p* < 0.001), with a difference of 11.2% (*p* = 0.100). AC correlated well (rho = 0.947, *p* < 0.001), with a mean difference of 9% (*p* = 0.070). Examples of disagreement between ground truth and model predictions are shown in Figure 6.

## 4. Discussion

This study demonstrates a deep learning method that provides rapid, end-to-end, pixel-wise segmentation of both vessel-specific CAC and AC in gated non-contrast cardiac CTs. To our knowledge, this is the first demonstrated application of such a model. The mode showed high pixel-level accuracy and CACS agreement to manual ground truth annotation. This was consistent across sex- and age-stratified subgroups. This facilitates the rapid throughput and reading of scans.

This potentiates clinically relevant consequences. The model is able to identify calcium on a pixel level, resulting in no informational resolution loss. Currently, commercially available CAC-scoring programs pool pixel values. Consequently, despite uniform scanning parameters, a patient may have different CACSs, and differing subsequent risk categorizations, when using different vendor-provided software [13]. A pixel-wise model averts this and allows future flexibility to adapt the output to meet a number of needs.

Firstly, there is the ability to automatically output CACS with high precision. Automated methods for this have previously been demonstrated, but not on a vessel-specific basis. de Vos et al. demonstrated a method that could directly output score-based risk category from cardiac or thoracic CTs [14]. This method predicted categories of risk, rather than a specific integer. Whilst being fast (<0.15 s per scan on a GPU) and accurate (cardiovascular risk categorization of 93%), it is noted that the model was not meant to provide pixel-level calcium identification, with a lower Dice score of 0.81, suggesting a loss in informational resolution. Similarly, other automated deep learning methods have been designed to output CACS, but with a loss of information at the pixel level [15,16,17,18].

Secondly, this facilitates the gleaning of more information from the standard cardiac CT. Whilst conventional scoring protocols for CAC detection have also used volume and mass, further prognostic imaging information such as density, diffusivity, plaque burden, regional distribution, progression, number of lesions, size, and shape can be obtained if pixel-wise information can be harnessed [1]. The current study may facilitate rapid analysis and detailed exploration into these parameters. Current conventional scoring methods have withstood the test of time due to parsimony at the expense of information loss. This study provides for the future development of more representative and intricate scoring systems that may be more time and labor efficient, but with higher information density.

Thirdly, this study allows the identification of prognostically significant AC, with potential incorporation into future risk prediction tools. It facilitates the further detailed study of relationships between CAC and AC and outcomes. A prior study has been able to identify coronary and extracoronary calcium on a voxel level, albeit on low-dose non-contrast chest CT scans [19]. In that study, the model was able to not only identify coronary calcium but was able to identify the coronary artery involved. Furthermore, AVC, TAC and mitral annular calcification were all identifiable, with Dice scores of 0.66–0.90. Those results portend avenues of expansion for the current study. Currently, non-contrast cardiac CTs are not used to evaluate CAC in patients with coronary stents, as contemporary software is unable to distinguish between CAC and metal coronary stents, and CAC quantification is inaccurate due to blooming artifact from the stent. However, further expansion of this algorithm’s use may include the recognition of coronary stents and accurate quantification of CAC correcting for the blooming artifact. This will require the requisite training datasets.

Finally, a deep learning model would reproduce the same result every time, lending consistency to image-based annotation and analysis, avoiding human reader fatiguability and error. During training and testing, a few model outputs identified annotation error by human readers that were later corrected (Figure 6). This raises the future possibility of a deep learning ‘second reader’ to be used in the practical workflow to minimize reader error.

This study and method carry inherent limitations. When compared to conventional clinical trials, the cohort size of this study was not as large. However, the dataset from 377 patients contains 24,507 images, numbers that are more than amply acceptable in size for medical image-based deep learning applications [20]. Although the present study is one of the largest deep learning-based demonstrations of vessel-specific CAC identification using non-contrast cardiac CTs that have been evaluated on a pixel basis, validation using larger cohorts is required. This shortcoming is reflected in Figure 5B, showing an underestimation bias of CAC when compared to the ground truth, with likely under-identification of CAC. It is possible that training on more varied images will reduce this bias. Secondly, the model was validated internally within the cohort. To address this, the study cohort was aggregated from multicenter registries and included a ‘hold-out’ validation set. The model was trained and tested on a randomized 80% of the patients, and never ‘saw’ the remaining 20% of patients until the final performance evaluation. Thirdly, this study had a high prevalence of high CAC and is not representative of the general population. Prior work has shown poorer prediction at higher rather than lower CAC values, whereas this model performed well in a high CAC cohort [21].

Concluding, a deep-learning model was able to rapidly and robustly identify vessel-specific CAC and AC. This was done with high speed, accuracy and agreement on both pixel-based and score-based levels, facilitating the expansion of its application into areas of research and clinical import.

## Figures and Tables

**Table 1:** Baseline characteristics.

Characteristic	Total
N	377
Age, years (SD)	64.2 ± 9.8
Female (%)	33.16
Diabetes Mellitus (%)	29.71
Dyslipidemia (%)	56.50
Hypertension (%)	64.19
Smoker (%)	32.36
LAD calcification (%)	74.87
RCA calcification (%)	55.82
LCx calcification (%)	57.41
Triple vessel calcification (%)	46.03
Aortic calcification (%)	85.41
CACS 0 (%)	11.39
CACS 1–99 (%)	11.39
CACS 100–399 (%)	24.05
CACS ≥ 400 (%)	53.16

LAD = left anterior descending, RCA = right coronary artery, LCx = left circumflex, CACS = coronary artery calcium score.

**Table 2:** Model performance.

Structure	Category	Dice Score		
		Median	Quartile (1st, 3rd)	*p*
Total coronary (LAD + RCA + LCx)	Overall	0.952	(0.921, 0.981)	-
	Male	0.948	(0.920, 0.981)	0.350
	Female	0.965	(0.933, 0.980)	
	Age < 65 years	0.950	(0.913, 0.981)	0.742
	Age ≥ 65 years	0.957	(0.930, 0.977)	
LAD	Overall	0.971	(0.930, 1.000)	-
	Male	0.963	(0.919, 1.000)	0.058
	Female	0.988	(0.968, 1.000)	
	Age < 65 years	0.970	(0.941, 0.999)	0.980
	Age ≥ 65 years	0.975	(0.911, 1.000)	
RCA	Overall	0.963	(0.889, 0.991)	-
	Male	0.951	(0.880, 1.000)	0.633
	Female	0.977	(0.923, 0.991)	
	Age < 65 years	0.964	(0.874, 0.999)	0.875
	Age ≥ 65 years	0.959	(0.899, 0.987)	
LCx	Overall	0.955	(0.894, 1.000)	-
	Male	0.954	(0.887, 1.000)	0.388
	Female	0.958	(0.942, 0.998)	
	Age < 65 years	0.954	(0.905, 0.999)	0.897
	Age ≥ 65 years	0.955	(0.887, 1.000)	
Aortic	Overall	0.832	(0.759, 0.897)	
	Male	0.802	(0.760, 0.905)	0.996
	Female	0.834	(0.764, 0.883)	
	Age < 65 years	0.833	(0.776, 0.933)	0.204
	Age ≥ 65 years	0.793	(0.756, 0.862)	

LAD = left anterior descending; RCA = right coronary artery; LCx = left circumflex; CACS = coronary artery calcium score.

## Data Availability

The data presented in this study are available on request from the corresponding author. The data are not publicly available due to privacy and ethical reasons.

## References

[1] Blaha M.J., Budoff M.J., Tota-Maharaj R.T., Dardari Z.A., Wong N.D., Kronmal R.A., Eng J., Post W.S., Blumenthal R.S., Nasir K. (2016). Improving the CAC Score by Addition of Regional Measures of Calcium Distribution: Multi-Ethnic Study of Atherosclerosis. JACC Cardiovasc. Imaging.

[2] Agatston A.S., Janowitz W.R., Hildner F.J., Zusmer N.R., Viamonte M., Detrano R. (1990). Quantification of coronary artery calcium using ultrafast computed tomography. J. Am. Coll. Cardiol..

[3] Shaw L.J., Min J.K., Nasir K., Xie J.X., Berman D.S., Miedema M.D., Whelton S.P., Dardari A.Z., Rozanski A., Rumberger J. (2018). Sex differences in calcified plaque and long-term cardiovascular mortality: Observations from the CAC Consortium. Eur. Heart J..

[4] Shaw L.J., Narula J., Chandrashekhar Y. (2015). The Never-Ending Story on Coronary Calcium: Is it predictive, punitive, or protective?. J. Am. Coll. Cardiol..

[5] Singh G., Al’Aref S.J., van Assen M., Kim T.S., Van Rosendael A., Kolli K.K., Dwivedi A., Maliakal G., Pandey M., Wang J. (2018). Machine learning in cardiac CT: Basic concepts and contemporary data. J. Cardiovasc. Comput. Tomogr..

[6] Hinton G. (2018). Deep Learning—A Technology with the Potential to Transform Health Care. JAMA.

[7] Rizvi A., Hartaigh B.Ó., Knaapen P., Leipsic J., Shaw L.J., Andreini D., Pontone G., Raman S., Khan M.A., Ridner M. (2016). Rationale and Design of the CREDENCE Trial: Computed TomogRaphic evaluation of atherosclerotic DEtermiNants of myocardial IsChEmia. BMC Cardiovasc. Disord..

[8] Chang H.-J., Lin F.Y., Lee S.-E., Andreini D., Bax J., Cademartiri F., Chinnaiyan K., Chow B.J., Conte E., Cury R.C. (2018). Coronary Atherosclerotic Precursors of Acute Coronary Syndromes. J. Am. Coll. Cardiol..

[9] Mark D.B., Berman D.S., Budoff M.J., Carr J.J., Gerber T.C., Hecht H.S., Hlatky M.A., Hodgson J.M., Lauer M.S., Miller J.M. (2010). ACCF/ACR/AHA/NASCI/SAIP/SCAI/SCCT 2010 Expert Consensus Document on Coronary Computed Tomographic Angiography: A report of the American College of Cardiology Foundation Task Force on Expert Consensus Documents. J. Am. Coll. Cardiol..

[10] Ronneberger O., Fischer P., Brox T. (2015). U-Net: Convolutional Networks for Biomedical Image Segmentation. Lecture Notes in Computer Science (Including Subseries Lecture Notes in Artificial Intelligence and Lecture Notes in Bioinformatics).

[11] He K., Zhang X., Ren S., Sun J. Delving Deep into Rectifiers: Surpassing Human-Level Performance on ImageNet Classification. Proceedings of the IEEE International Conference on Computer.

[12] Dice L.R. (1945). Measures of the Amount of Ecologic Association between Species. Ecology.

[13] Weininger M., Ritz K.S., Schoepf U.J., Flohr T.G., Vliegenthart R., Costello P., Hahn D., Beissert M. (2012). Interplatform Reproducibility of CT Coronary Calcium Scoring Software. Radiology.

[14] De Vos B.D., Wolterink J.M., Leiner T., De Jong P.A., Lessmann N., Leiner I. (2019). Direct Automatic Coronary Calcium Scoring in Cardiac and Chest CT. IEEE Trans. Med. Imaging.

[15] Wolterink J.M., Leiner T., Takx R.A.P., Viergever M.A., Isgum I. (2015). Automatic Coronary Calcium Scoring in Non-Contrast-Enhanced ECG-Triggered Cardiac CT with Ambiguity Detection. IEEE Trans. Med. Imaging.

[16] Isgum I., Prokop M., Niemeijer M., Viergever M.A., van Ginneken B. (2012). Automatic Coronary Calcium Scoring in Low-Dose Chest Computed Tomography. IEEE Trans. Med. Imaging.

[17] Cano-Espinosa C., González G., Washko G.R., Cazorla M., Estépar S.J.R. (2018). Automated Agatston score computation in non-ECG gated CT scans using deep learning. Proc. SPIE.

[18] Shadmi R., Mazo V., Bregman-Amitai O., Elnekave E. (2018). Fully-convolutional deep-learning based system for coronary calcium score prediction from non-contrast chest CT. Proceedings of the 2018 IEEE 15th International Symposium on Biomedical Imaging (ISBI).

[19] Lessmann N., van Ginneken B., Zreik M., de Jong P.A., de Vos B.D., Viergever M.A., Išgum I. (2018). Automatic Calcium Scoring in Low-Dose Chest CT Using Deep Neural Networks with Dilated Convolutions. IEEE Trans. Med. Imaging.

[20] Cho J., Lee K., Shin E., Choy G., Do S. (2015). How much data is needed to train a medical image deep learning system to achieve necessary high accuracy?. arXiv.

[21] Santini G., della Latta D., Martini N., Valvano G., Gori A., Ripoli A., Susini C.L., Landini L., Chiappino D. (2017). An automatic deep learning approach for coronary artery calcium segmentation. Proceedings of the EMBEC & NBC 2017 Joint Conference of the European Medical and Biological Engineering Conference (EMBEC) and the Nordic-Baltic Conference on Biomedical Engineering and Medical Physics (NBC).

